# Utilization of dexrazoxane in patients treated with doxorubicin: a retrospective, propensity matched analysis of cardiac function and toxicity

**DOI:** 10.3389/fonc.2025.1621409

**Published:** 2025-07-16

**Authors:** Zachary M. Neiman, Carlo S. Legasto, Alan K. Chin, Tiffany Guan, Brian C. Schulte

**Affiliations:** ^1^ School of Medicine, University of California San Francisco, San Francisco, CA, United States; ^2^ Department of Pharmaceutical Services, University of California San Francisco, San Francisco, CA, United States; ^3^ Helen Diller Family Comprehensive Cancer Center, University of California San Francisco, San Francisco, CA, United States; ^4^ Division of Hematology and Oncology, Department of Medicine, University of California San Francisco, San Francisco, CA, United States

**Keywords:** oncology, dexrazoxane, doxorubicin, anemia, neutropenia, chemotherapy, ejection fraction, cardiotoxicity

## Abstract

**Introduction:**

Dexrazoxane (DZR) has been used to prevent cardiotoxicity from doxorubicin (DOX), particularly in younger patients with cancer and those with pre-existing cardiac dysfunction. Herein, we sought to further define the role of DZR by evaluating its capacity to mitigate cardiotoxicity in patients actively receiving DOX, while also assessing concomitant toxicities.

**Materials and methods:**

We conducted a retrospective, propensity-matched cohort study at a single academic center, comparing outcomes between patients treated with DZR plus DOX and those who received DOX alone. Patients were matched by age, sex, and cumulative lifetime dose of DOX. Cardiotoxicity was assessed as the change in ejection fraction (EF) during and after treatment. To evaluate associations between DZR and other toxicities, we utilized the Common Terminology Criteria for Adverse Events, Version 5 (CTCAE).

**Results:**

A total of 152 patients were included across both groups. The DOX alone and DOX + DZR groups had median ages of 36 and 28 (ranges 18–68 and 18–69), with median cumulative DOX doses of 375 mg/m^2^ (ranges 75–525 and 75–600), respectively. Patients were followed up with their last measured EF at a median of −3 and 18.5 days after their final DOX dose, respectively. The median change in EF was −2% in the DOX alone group and −0.7% in the DOX + DZR group (p = 0.9174). Grade 4 anemia occurred in 16 patients in the DOX alone group and in 41 patients in the DOX + DZR group (p = 0.0002). Similarly, grade 4 neutropenia was observed in 15 and 50 patients, respectively (p = 0.0013).

**Discussion:**

The addition of DZR to DOX did not result in a statistically significant change in EF during the treatment window. Given the limitations of the dataset, this may suggest a lack of substantial immediate benefit from the co-administration of DZR with DOX. An increased rate of high-grade neutropenia and anemia was observed in patients receiving the combination, although this may be due to confounding factors. Further analysis is warranted, ideally through larger multi-institutional or prospective studies.

## Introduction

Doxorubicin (DOX) is a commonly administered cytotoxic chemotherapeutic agent approved for multiple oncologic indications (1). While effective, DOX is associated with several toxicities, including cardiotoxicity, secondary malignancies, and myelosuppression (1). Dexrazoxane (DZR) is FDA approved for preventing DOX-induced cardiotoxicity in patients who have reached a cumulative lifetime dose greater than 300 mg/m^2^ (2–4). DZR has been studied in patients with breast cancers, sarcomas, and small cell lung cancer (5), and has demonstrated cardioprotective effects (6, 7). While complex, the mechanism of DZR is thought to involve iron binding and displacement of DOX, leading to reduced generation of reactive oxygen species (8, 9).

Early questions on the co-administration of DZR and DOX suggested no reduction in efficacy (8). A randomized phase III study in patients with breast cancer showed a reduction in cardiac events with DZR (13% from 39%) and an unchanged response rate (35% for both groups) (10). An interim analysis of a phase II single-arm noninferiority trial in advanced or metastatic soft tissue sarcoma reported a progression-free survival of 8.4 months, compared to a historical survival of 4.6 months (11). Given the suggested preservation of efficacy, DZR has seen increasing use in patients with malignancies.

Herein, we aimed to further characterize the toxicities in patients who did or did not receive DZR in addition to DOX by retrospectively evaluating propensity-matched cohorts in real-world settings. Cardiotoxicity was measured by assessing changes in ejection fraction (EF) over the course of treatment. We also assessed the incidence of common toxicities, specifically anemia and neutropenia, which have been reported to increase with the addition of DZR to DOX (anemia: from 73% to 86%; neutropenia: from 31% to 45%) (12). We leveraged existing variations in drug dosing and scheduling to better understand which regimens may be most effective in preventing cardiotoxicity.

## Materials and methods

### Data sources

Patient data were initially queried, followed by chart review using the UCSF electronic health record (EHR). Patient data were collected and organized using REDCap electronic data capture tools hosted at UCSF (13). The Institutional Review Board of the University of California, San Francisco, approved this research.

### Patient selection

Patients included in this study had a history of malignancy, were 18 years of age or older, and had previously received DOX or a combination of DOX and DZR. Patients were treated at UCSF Health between 1 January 2011 and 31 July 2024. The clinical rationale for adding DZR to DOX was not assessed. A total of 76 patients who received both DOX and DZR were identified and propensity-matched by age, sex, and dose to 76 matched patients who received DOX alone. Propensity scores were calculated using logistic regression, with age, sex, and dose as covariates. Patients from the DOX/DZR and DOX groups were subsequently matched based on the closest propensity scores. These calculations were performed in Excel using the Solver add-in (14). Patient data were populated through 1 January 2025.

### Patient characteristics

Patient characteristics included age, sex, and race, as listed in the EHR. Diagnoses were categorized as sarcoma or breast cancer based on initial data extraction and further refined through manual chart review (**Supplementary Table 1**). Additional patient characteristics derived from chart review included smoking history and pack-years (if available); common comorbidities (diabetes, hypertension, coronary artery disease, dyslipidemia, chronic obstructive pulmonary disease, asthma, stroke, gastrointestinal disease, chronic kidney disease, and preexisting heart failure); whether the patient was established with a cardiologist; and use of cardioprotective medications (statins, angiotensin-converting enzyme inhibitors, angiotensin receptor blockers, or beta blockers). Dates of death were recorded, and time from the first DOX dose to death was calculated for deceased patients.

### Outcomes

To evaluate cardiotoxicity, the dates and doses of doxorubicin and dexrazoxane were collected. The closest EF measurement (value and date) prior to the first dose of DOX was collected as the baseline. Each subsequent echocardiogram was reviewed for EF. Follow up duration was recorded as the number of days from the last DOX dose to the last available EF. Lifetime cumulative doses of DOX and DZR were calculated. Variances in administration of each agent were also recorded. Additional variables included the time and cumulative lifetime dose of DOX at the time of first DZR administration.

To evaluate anemia and neutropenia, the lowest hemoglobin and neutrophil levels observed during treatment or within four weeks after a dose were recorded. These values were then graded according to CTCAE Version 5 (15)

The median change in EF was calculated for both the single-agent DOX group and the DOX plus DZR combination group. To better evaluate the degree of EF change, quantitative changes were primarily used for comparison, rather than categorizing changes based on predefined EF thresholds. An additional analysis was performed to evaluate the dosing of the first DOX cycle (which was largely consistent with later cycles): one dose over one day, two doses over two days, and three doses over three days. Among patients who received at least 300 mg/m^2^ of cumulative lifetime DOX, the median EF change was calculated for the following groups: [1] DZR initiated on cycle 1, day 1 of DOX; [2] DZR started after cycle 1 but prior to 300 mg/m^2^ of DOX; and [3] DZR administered at or after 300 mg/m^2^. For evaluating differences in the DZR: DOX dosing ratio, the DOX + DZR group was subdivided into three groups: patients whose EF did not decrease, those with a decrease of less than 10%, and those with a decrease of 10% or more. The median change in EF was calculated for each subgroup, followed by determination of the median lifetime DZR: DOX ratios.

### Statistical analysis

Descriptive statistics were used to summarize the results. When counts were reported, corresponding percentages were also provided. When medians were calculated, standard deviations (SDs) and ranges were also reported. A two-tailed Student’s t-test was used to calculate p-values when comparing two groups with equal sample sizes and similar variances. Welch’s ANOVA was used to calculate p-values when comparing three groups with unequal sample sizes. Welch’s t-test was used when ANOVA could not be performed due to within-group degrees of freedom being less than one, resulting from insufficient sample size. The chi-square test was used to calculate p-values when comparing counts across different categories. A significant level (alpha) of 0.05 was used. All statistical analyses were performed using Microsoft Excel (14).

## Results

### Patient characteristics

The median age was 36 for the DOX alone group and 28 years for the DOX + DZR combination group (**Table 1**). Both groups included 45 male 45 and 31 female patients. A similar number of patients in both groups had documented smoking histories (17 in the DOX group and 18 in the DOX + DZR group). Among patients with documented pack-year data, the median (standard deviation [SD]) pack-years were 10 (9.3) in the DOX group and 3.8 (3.4) in the DOX + DZR group. A total of 13 patients in the DOX group and 20 in the DOX + DZR group were established with a cardiologist.

**Table 1:** Patient characteristics.

Characteristics	Breakdown	DOX alone group	DOX + DZR group
Total number		76	76
Age		36 (13.7; 18–68)	28 (15.6; 18–69)
Sex	Male	45 (59.2)	45 (59.2)
	Female	31 (40.8)	31 (40.8)
Race	Asian	11 (14.5)	9 (11.8)
	Black or African American	1 (1.3)	9 (11.8)
	White	37 (48.7)	39 (51.3)
	Other	27 (35.5)	19 (25)
Diagnosis (specifics in supplemental)	Sarcoma	67 (88.2)	75 (98.7)
	Breast cancer	9 (11.8)	1 (1.3)
Smoking history	Smoked count; pack years median (SD)	17 with history, 11 with pack years; 10 (9.3; 0.3–26)	18 with history, 15 with pack-years; 3.8 (3.4; 0.02–10)
	Never smoked	59 (77.6)	58 (76.3)
Comorbidities	Diabetes	6 (7.9)	2 (2.6)
	Hypertension	15 (19.7)	6 (7.9)
	CAD	1 (1.3)	1(1.3)
	Dyslipidemia	9 (11.8)	3 (3.9)
	COPD	0 (0)	0 (0)
	Asthma	7 (9.2)	5 (6.6)
	Stroke	2 (2.6)	0 (0)
	GI disease	7 (9.2)	4 (5.3)
	CKD	0 (0)	1(1.3)
	HF—preexisting	0 (0)	1(1.3)
Established with cardiologist		13 (17.1)	20 (26.3)
Concomitant cardioprotective medications (specifics in supplemental)	Statin	7	4
	Acei or ARB	4	3
	Beta Blocker	9	3
Death	Number died	21 (27.6)	27 (35.5)
	Of those, days alive after first DOX dose	840 (759; 134–2,932)	399 (402; 35–1,624)

Shown as median (SD; range); or count (percentage). CAD, coronary artery disease; COPD, chronic obstructive pulmonary disease; GI, gastrointestinal; CKD, chronic kidney disease; HF, heart failure.

### Toxicities

There were 16 patients with CTCAE Grade 4 anemia in the DOX alone group and 41 in the DOX + DZR groups (p = 0.0002) (**Table 2**). For CTCAE Grade 4 neutropenia, 15 patients were in the DOX alone group and 50 in the DOX + DZR group (p = 0.0013).

**Table 2:** Patient toxicities: highest CTCAE grade during treatment or up to 4 weeks after a dose.

Toxicities	Breakdown	DOX alone group	DOX + DZR group
Anemia	Grade 1	8 (10.5)	1 (1.3)
	Grade 2	25 (32.9)	15 (19.7)
	Grade 3	24 (31.6)	19 (25)
	Grade 4	16 (21.1)	41 (53.9)
	Chi-square p-value	0.0002	
Neutropenia	Grade 1	6 (7.9)	3 (3.9)
	Grade 2	6 (7.9)	3 (3.9)
	Grade 3	7 (9.2)	4 (5.3)
	Grade 4	15 (19.7)	50 (65.8)
	Chi-square p-value	0.0013	

Shown as count (percentage).

### DOX and DZR administration

The median lifetime dose of DOX for each group was 375 mg/m^2^ (**Table 3**). The median lifetime dose of DZR was 2,250 mg/m^2^ in the DOX + DZR group. In the DOX alone group, 10 patients received DOX as a single dose (bolus or continuous infusion) over one day; eight received two doses over two days; 58 received three doses over three days; and 62 received DOX as continuous 24-hour infusions. In comparison, in the DOX + DZR group, eight patients received DOX as a single dose over one day; 40 received two doses divided over two days; 28 received three doses divided over three days; and 30 received continuous 24-hour infusions. In the DOX + DZR group, the median number of days from the first DOX dose to DZR initiation was 0, with a SD of 171 (range 0–930). The median cumulative DOX dose at the time of first DZR administration was 0 mg/m^2^, with a SD of 147 (range 0–510).

**Table 3:** Statistics of DOX and DZR administration, and EF follow up.

Statistics	Breakdown	DOX alone group	DOX + DZR group
Days of follow up (days from last DOX dose to last EF)		−3 (604; −250–3,234)	18.5 (614; −135–3,682)
Lifetime DOX mg/m^2^		375 (110.9; 75–525)	375 (124.1; 75–600)
Lifetime DZR mg/m^2^		0	2,250 (1,306; 225–4,500)
DOX administration schedule	1 dose over 1 day	10 (13.2)	8 (10.5)
	2 doses over 2 days	8 (10.5)	40 (52.6)
	3 doses over 3 days	58 (76.3)	28 (36.8)
DZR administration schedule	1 dose over 1 day	n/a	13 (17.1)
	2 doses over 2 days	n/a	37 (48.7)
	4 doses over 2 days	n/a	1 (1.3)
	6 doses over 3 days	n/a	25 (32.9)
Median number of days between cycles		28 (13.2; 13–112.5)	28.5 (11.4; 15–89.5)
Number of days between the first DOX cycle and initiation of DZR		n/a	0 (171; 0–930)
Cumulative dose of DOX administered at DZR initiation		n/a	0 (147; 0–510)
DOX given continuously over 24 hours or as a push	Continuous	62 (81.6)	30 (39.5)
	Push	14 (18.4)	46 (60.5)

Shown as median (SD; range); or count (percentage).

### Change in EF

**Figure 1:**
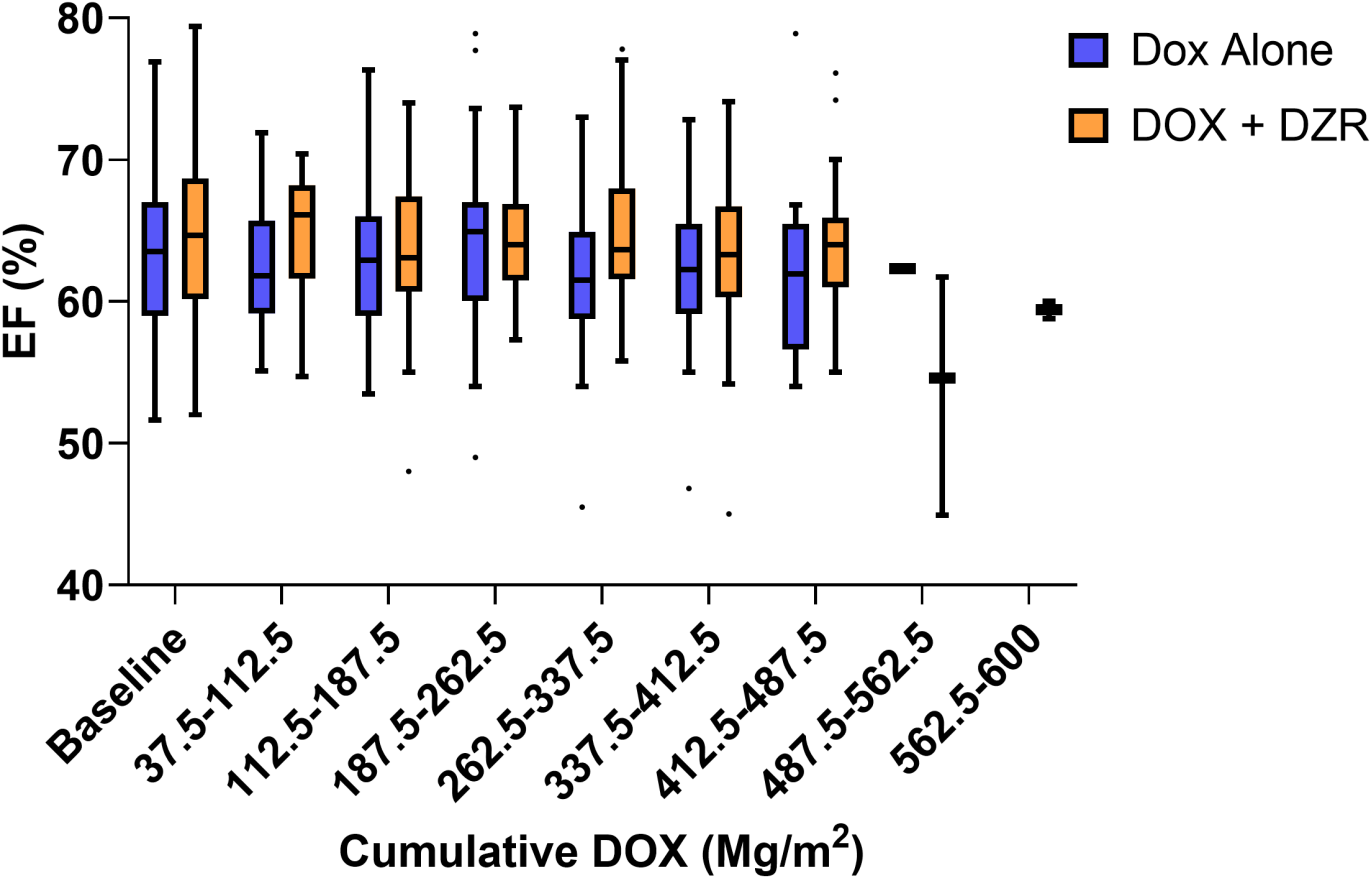
Ejection fraction (EF, %) by cumulative dose of doxorubicin (DOX) throughout treatment course. Box and whisker plots with outliers illustrate the distribution of EF values at various cumulative DOX doses. Both the DOX alone and DOX combined with dexrazoxane (DZR) groups are represented.

**Figure 2:**
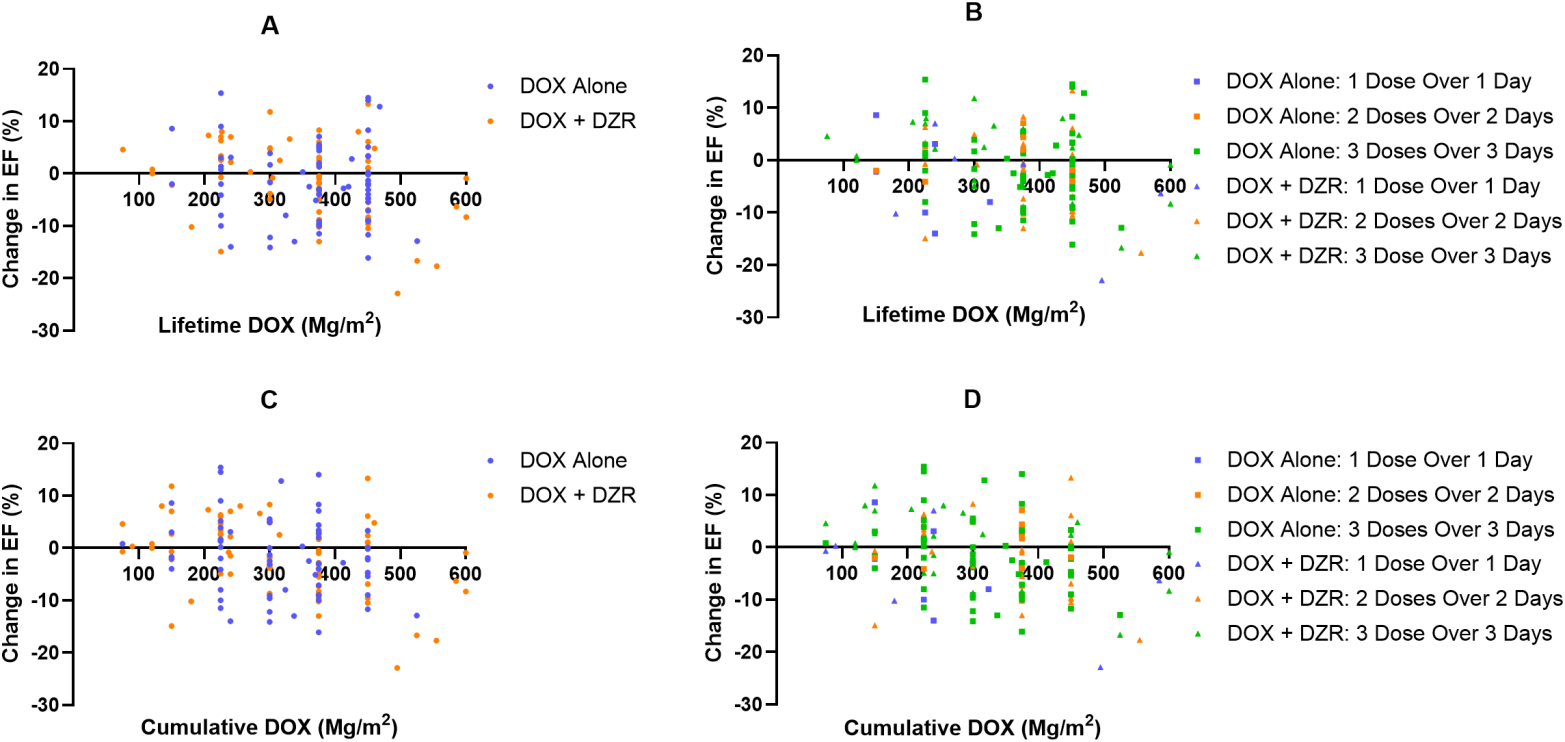
Change in ejection fraction (EF, %), as a function of doxorubicin (DOX) for both the DOX alone and DOX combined with dexrazoxane (DZR) groups. **(A)** Change in EF by lifetime cumulative DOX dose, categorized by treatment group (DOX alone vs. DOX + DZR). **(B)** Change in EF by lifetime cumulative DOX dose, categorized by DOX dosing schedule. **(C)** Change in EF by cumulative DOX at the time of the last EF measurement, categorized by DOX alone or DOX + DZR. **(D)** Change in EF by cumulative DOX at the time of the last EF measurement, categorized by DOX dosing schedule.

The median change in EF was −2% for the DOX alone group and −0.7% for the DOX + DZR group (p = 0.9174) (**Table 4**; **Figures 1**, **2A, C**).

**Table 4:** Primary outcomes.

Outcome	Breakdown	DOX alone group	DOX + DZR group
Overall: median change in EF (%) (**Figures 2A, C**)		−2 (7.3; −16.1–15.4)	−0.7 (7.2; −22.9–13.3)
	Student’s t-test p-value	0.9174	
Scheduling: median change in EF (%) for different scheduling (**Figures 2B, D**)	1 dose over 1 day	−8 (9.5; −14–8.6)	−4.3 (8.9; −22.9–7)
	2 doses over 2 days	−1.9 (4.4; −4.1–7.1)	−0.7 (7.0; −17.7–13.3)
	3 doses over 3 days	−2.2 (7.4; −16.1–15.4)	1.5 (6.5; −16.7–11.8)
	Student’s t-test p-values (between 1 and 2 days, between 2 and 3 days, and between 1 and 3 days)	0.3897 0.3305 0.6216	0.3313 0.1993 0.1244
DZR at different starting points of DOX: for those with lifetime DOX ≥300 mg/m^2^, median change in EF (**Figure 3A**)	DZR given at start (Cumulative DOX at 0 mg/m^2^)	n/a	−0.75 (5.8; −10.5–13.3)
	DZR starts being given when DOX <300 mg/m^2^		−4.2 (7.3; −13–11.8)
	DZR started being given when DOX ≥300 mg/m^2^		−8.3 (8.7; −22–4.8)
	Welch’s ANOVA p-value		0.1583
Dosing: median DZR: DOX ratio by different changes in EF (**Figure 4A**)	EF does not decrease	n/a	10 (2.9; 2.5–10.5)
	EF decreases by less than 10%		9.2 (3.3; 1.7–10)
	EF decreases by 10% or more		3.7 (3.8; 0.4–10)
	Welch’s ANOVA p-value		0.0037

Shown as median (SD; range) or p-value.

In the DOX alone group, patients who received their first cycle as one dose over one day, two doses over two days, and three doses over three days had median EF changes of −8%, −1.9%, and −2.2%, respectively (p = 0.3897, 0.3305, and 0.6216) (**Table 4**; **Figures 2B, D**). In the DOX + DZR group, the corresponding median EF changes were -4.3%, -0.7%, and 1.5% (p = 0.3313, 0.1993, and 0.1244).

**Figure 3:**
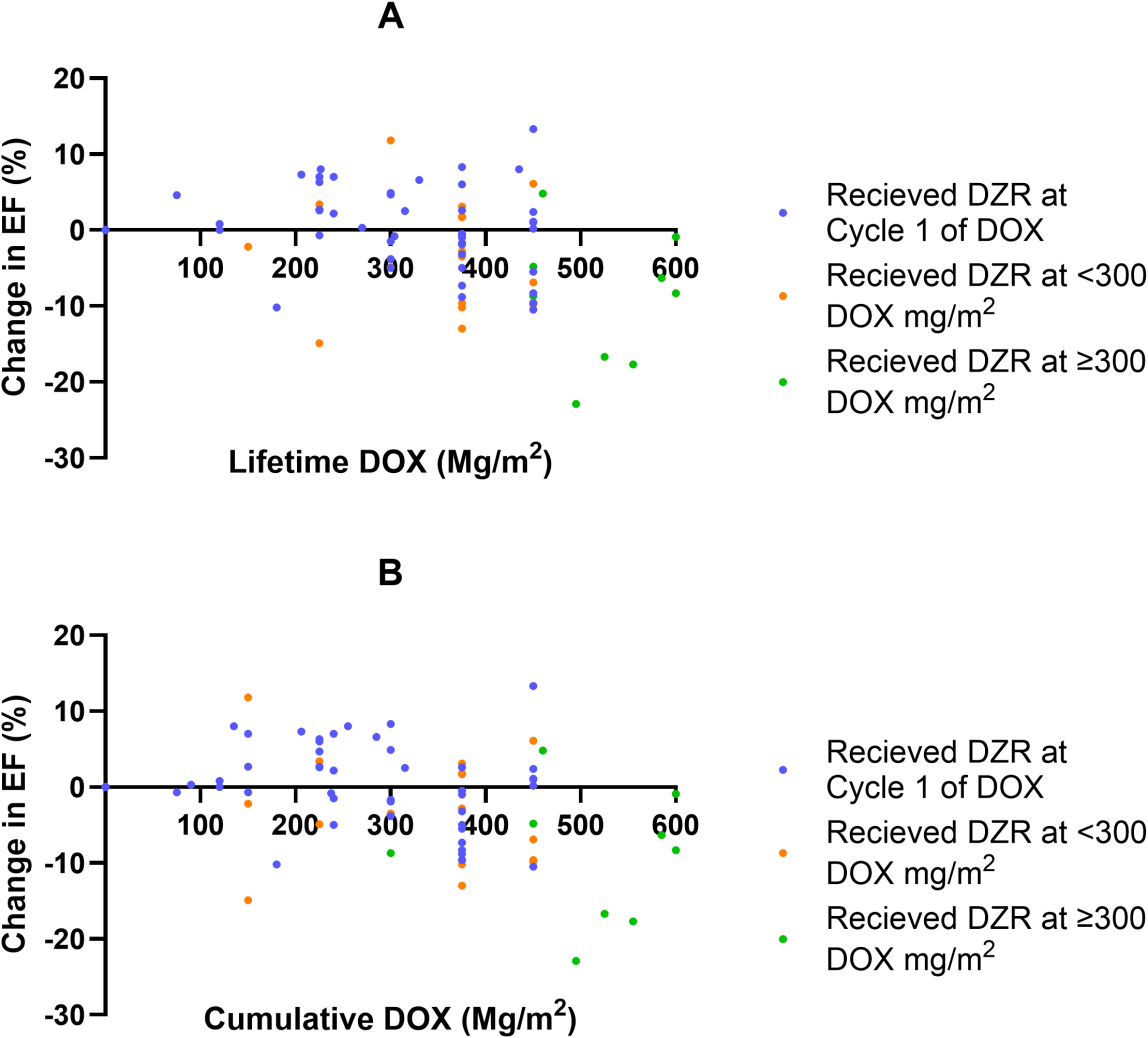
Change in ejection fraction (EF, %) as a function of doxorubicin (DOX) in the DOX + dexrazoxane (DZR) group. **(A)** Change in EF by lifetime cumulative DOX dose, categorized by cumulative DOX at the time of DZR initiation. **(B)** Change in EF by cumulative DOX at the time of the last EF measurement, categorized by cumulative DOX at DZR initiation.

Among patients in the DOX + DZR group who received at least 300 mg/m^2^ cumulative DOX, the median EF change was -0.75% for those who initiated DZR at a DOX dose of 0 mg/m^2^ (**Figure 3A**). Those who began DZR at a cumulative DOX dose between 0 mg/m^2^ and 300 mg/m^2^ experienced a median EF change of −4.2%. Those who started DZR after reaching 300 mg/m^2^ of DOX had a median EF change of −8.3% (p = 0.1583). A similar trend was observed when using the cumulative DOX dose at the time of the last EF measurement rather than lifetime DOX, with corresponding median EF changes of −1.35%, −5.2%, and −8.3%, respectively (**Table 5**; **Figure 3B**).

**Table 5:** Primary outcomes with cumulative dose at last EF measured rather than lifetime dose.

Outcome	Breakdown	DOX alone group	DOX + DZR group
DZR at different starting points of DOX: for those with total DOX ≥300 mg/m^2^ at last EF taken, median change in EF (**Figure 3B**)	DZR given at start (Cumulative DOX at 0 mg/m^2^)	n/a	−1.35 (5.9; −10.5–13.3)
	DZR starts being given when DOX <300 mg/m^2^		−5.2 (6.3; −13–6.1)
	DZR started being given when DOX ≥300 mg/m^2^		−8.3 (8.7; −22.9–4.8)
	Welch’s ANOVA p-value		0.1848
Dosing: median DZR: DOX ratio by different changes in EF (**Figure 4B**)	EF does not decrease	n/a	10 (2.8; 2.5–10.5)
	EF decreases by less than 10%		9 (3.1; 1.7–10)
	EF decreases by 10% or more		4 (3.8; 0.4–10)
	Welch’s ANOVA p-value		0.0099

Shown as median (SD; range) or p-value.

**Figure 4:**
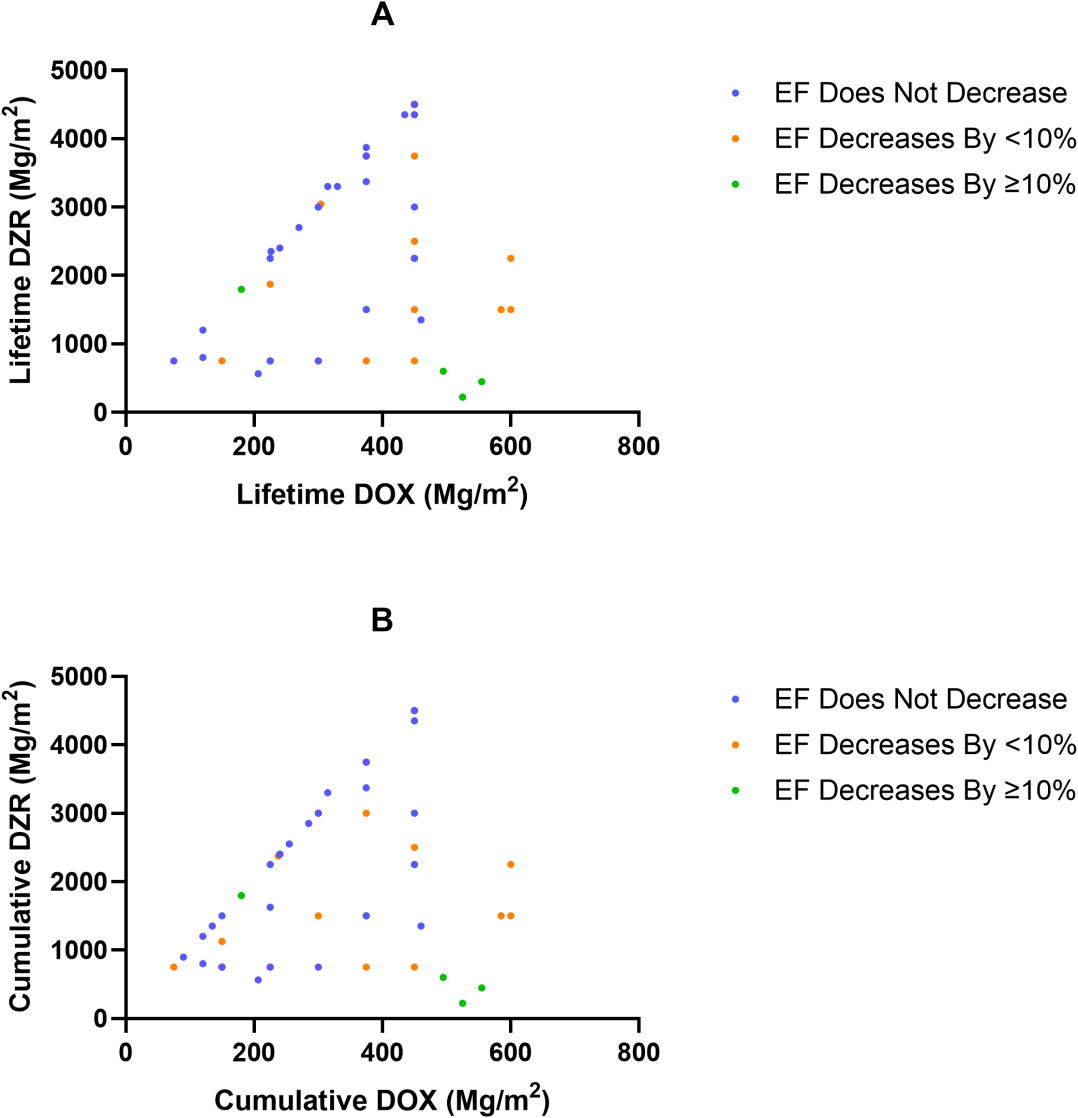
Lifetime dexrazoxane (DZR) exposure as a function of doxorubicin (DOX), categorized by ejection fraction (EF, %) change in the DOX + DZR group. **(A)** Lifetime DZR dose as a function of lifetime DOX dose, categorized by EF change. **(B)** Cumulative DZR dose as a function of cumulative DOX at the time of the last EF measurement, categorized by EF change.

Among patients in the DOX + DZR group, those without a decline in EF had a median DZR: DOX ratio of 10; those with an EF decline of less than 10% had a median ratio of 9.2; and those with a decline of 10% or more had a significantly lower median ratio of 3.7 (p = 0.0037) (**Table 4**; **Figure 4A**). Similar findings were observed when using the cumulative DOX dose at the time of the last EF measurement instead of lifetime DOX, with corresponding median DZR: DOX ratios of 10, 9, and 4, respectively (**Table 5**; **Figure 4B**).

## Discussion

In this propensity-matched retrospective analysis of patients who received DOX alone or in combination with DZR at a single institution, there was no significant difference in the incidence of EF reduction during the treatment window. However, the analysis revealed a higher incidence of Grade 4 anemia and neutropenia, as defined by CTCAE. This study has several limitations: it is retrospective, conducted at a single site, and includes relatively short follow-up for cardiac events, which is notable as chronic doxorubicin toxicity can manifest years after administration (16). Additionally, more patients in the DOX + DZR group received DOX as bolus rather than continuous infusions over 24 h–72 h, and continuous infusion has been associated with reduced cardiotoxicity (17). Propensity matching helped reduce differences related to age, sex, and cumulative dose; however, variables such as prior treatment history, performance status, and concomitant chemotherapeutic agents were not included in the regression, potentially limiting causal inference. The groups were similar in the number of patients with a smoking history, although the median recorded pack-years were higher in the DOX alone group.

The long-term effects of anthracycline exposure have been well described (18, 19), although long-term patient follow-up remains challenging, particularly as clinical practices evolve. Nonetheless, this must be considered in the clinical context of conditions which frequently have a limited overall life expectancy, such as advanced solid tumors. The inclusion of both curable and incurable diagnoses may limit interpretability. Nonetheless, given the relatively similar prior and concurrent chemotherapy regimens between groups (**Supplementary Table 1**), including a similar number classified as high-risk for febrile neutropenia by NCCN criteria (20), the substantially higher rates of Grade 4 anemia and neutropenia in the combination group may be meaningful. This finding may be confounded by concomitant agents and chemotherapy regimens, although it was also observed in prior analyses (12, 21). Although DZR is known to contribute to chemotherapy-induced myelosuppression, the underlying mechanism remains unclear and warrants further investigation.

Differences exist in the administration of DZR and DOX, and our study offers insight into their co-administration. Administration also varies in whether DOX and DZR are delivered over one day or multiple days (22). Patients who receive DOX as a single dose over one day per cycle appeared to experience a slightly greater decrease in EF compared to those who received two or three doses over multiple days. This may suggest a modest benefit to administering DOX over multiple days, although the differences were not significantly significant.

FDA labeling recommends initiating DZR once the cumulative DOX dose reaches 300 mg/m^2^, although some guidelines consider starting at 250 mg/m^2–^300 mg/m^2^ to be reasonable (2–4, 23). This aligns with the understanding that the risk of DOX-induced heart failure increases exponentially as the dose exceeds 300 mg/m^2^ (24). The cardioprotective effect of DZR was demonstrated by a 3%incidence of CHF compared to 22% at DOX doses exceeding 300 mg/m^2^ (25). In our study, comparisons of different starting DZR initiation points within the DOX + DZR group did not yield statistically significant differences during the treatment window. However, a modest trend toward better-preserved EFs was observed when DZR was initiated earlier, suggesting that prospective studies may be warranted to further evaluate the benefits of starting DZR prior to reaching a cumulative DOX dose of 300 mg/m^2^.

FDA labeling also recommends administering DZR at a DZR: DOX ratio of 10:1 (3). Most studies and clinical practice maintain this 10:1 ratio (2, 4), although early studies have explored higher 20:1 ratios (26). An animal study identified an optimal dose ratio between 10:1 and 20:1 (27), although it remains unclear whether the commonly used 10:1 dose ratio is ideal in humans. In our study, the groups with EFs that did not decrease, decreased by less than 10%, or decreased by 10% or more showed statistically significant differences in lifetime DZR: DOX ratios. A ratio close to 10 was associated with EFs that either remained stable or declined by less than 10%, further supporting the use of a 10:1 DZR: DOX ratio. However, a limitation is that, unlike the comparison between the DOX alone with DOX + DZR groups, these subgroups were not matched for age and sex. In addition, the study was limited to pre-existing DZR: DOX ratios and was therefore not randomized. Future studies may be warranted to evaluate whether doses exceeding the 10:1 ratio enhance cardioprotection.

Given the increased risk of anemia and neutropenia associated with the DOX + DZR combination, there may be renewed interest in identifying a DZR dose that reduces cardiotoxicity while minimizing hematologic toxicity. This may be particularly relevant for patients with limited life expectancy and advanced disease, as the short-term risk of cardiotoxicity appears to be low. Further investigation is needed to guide clinicians on the timing of DZR use—whether upfront or delayed—particularly in relation to long-term outcomes. Overall, before initiating DZR, clinicians should carefully weigh its cardioprotective benefits against the risks of anemia and neutropenia, in the context of treatment goals.

## Data Availability

The datasets presented in this article are not readily available because primary data utilized in this analysis included PHI under the direction of the University of California San Francisco Institutional Review Board. Requests to access the datasets should be directed to zachary.neiman@ucsf.edu.
