# Supplementary material for: Utilization of dexrazoxane in patients treated with doxorubicin: a retrospective, propensity matched analysis of cardiac function and toxicity
Source: Front Oncol. 2025 Jul 16;15:1621409. doi: 10.3389/fonc.2025.1621409 (PMC12308200; doi:10.3389/fonc.2025.1621409)
Supplement: Supplementary file 1 [file Table1.docx]

**Supplemental Table 1.** **Breakdown of patient cancer diagnoses, concomitant cardioprotective medications,** **and** **chemotherapies / systemic treatments.**

|  | Breakdown | DOX Alone Group | DOX + DZR Group |
| --- | --- | --- | --- |
| Diagnosis | Osteosarcoma | 11 (14.5) | 19 (25) |
|  | Ewing sarcoma | 5 (6.6) | 14 (18) |
|  | Synovial sarcoma | 11 (14.5) | 7 (9.2) |
|  | Pleomorphic sarcoma | 6 (7.9) | 12 (16) |
|  | Leiomyosarcoma | 6 (7.9) | 5 (6.6) |
|  | Spindle cell carcinoma | 8 (10.5) | 0 (0) |
|  | IDC | 5 (6.6) | 0 (0) |
|  | Rhabdomyosarcoma | 0 (0) | 5 (6.6) |
|  | Chondrosarcoma | 4 (5.3) | 0 (0) |
|  | Round cell sarcoma | 3 (3.9) | 1 (1.3) |
|  | Liposarcoma | 2 (2.6) | 2 (2.6) |
|  | Epithelioid sarcoma | 2 (2.6) | 1 (1.3) |
|  | Myxofibrosarcoma | 2 (2.6) | 1 (1.3) |
|  | Embryonal sarcoma | 2 (2.6) | 1 (1.3) |
|  | Intimal sarcoma | 0 (0) | 3 (3.9) |
|  | DCIS | 2 (2.6) | 0 (0) |
|  | Angiosarcoma | 2 (2.6) | 0 (0) |
|  | Other (one of each): | Desmoid / sarcoma, uterine carcinosarcoma, ILC,  malignant peripheral nerve sheath tumor, malignant phyllodes tumor | Angiomatoid fibrous histiocytoma, endometrial stroma sarcoma, invasive lobular carcinoma, small cell ovarian cancer, gastrointestinal neuroectodermal tumor |
| Concomitant cardioprotective medications | Statin | 5 atorvastatin, 2 rosuvastatin | 4 atorvastatin |
|  | Angiotensin-converting enzyme inhibitor or angiotensin receptor blocker | 1 of each: losartan, valsartan, enalapril, lisinopril | 2 lisinopril, 1 valsartan |
|  | Beta Blocker | 3 metoprolol tartrate, 3 carvedilol, 1 of each: metoprolol succinate,  atenolol,  bisoprolol) | 1 of each: nadolol, metoprolol tartrate, carvedilol |
| Chemotherapies / systemic treatments had before or during doxorubicin administration | AIM (ifosfamide and doxorubicin) | 37 (48.7) | 22 (28.9) |
|  | Cisplatin and doxorubicin | 11 (14.5) | 10 (13.2) |
|  | VDC/IE (vincristine, doxorubicin, and cyclophosphamide / ifosfamide and etoposide) | 9 (11.8) | 11 (14.5) |
|  | VAC (vincristine, doxorubicin, and cyclophosphamide) | 2 (2.6) | 7 (9.2) |
|  | Cisplatin, doxorubicin and MTX (methotrexate) | 2 (2.6) | 6 (7.9) |
|  | AIM + monocolonal antibody | 0 (0) | 4 (5.3) |
|  | Cyclophosphamide, doxorubicin, paclitaxel, and monoclonal antibody | 3 (3.9) | 1 (1.3) |
|  | Gemcitabine, taxotere and doxorubicin | 1 (1.3) | 3 (3.9) |
|  | Doxorubicin | 1 (1.3) | 2 (2.6) |
|  | AIM and VAC | 1 (1.3) | 2 (2.6) |
|  | Doxorobucin + MTX | 0 (0) | 2 (2.6) |
|  | Gemcitabine, taxotere, doxorubicin, and alkylating agent | 0 (0) | 2 (2.6) |
|  | Doxorubicin and alkylating agent | 2 (2.6) | 0 (0) |
|  | Doxorubicin, cytoxan, and taxol/herceptin/perjeta | 2 (2.6) | 0 (0) |
|  | Cyclophosphamide, doxorubicin, and taxol | 2 (2.6) | 0 (0) |
|  | One of each: | Doxorubicin and cyclophosphamide; cyclophosphamide, doxorubicin, and monoclonal antibody; VIDE (vincristine, ifosfamide, etoposide, adriamycin). | VPCBAE (vinblastine, cisplatin, cyclophosphamide, bleomycin, doxorubicin and etoposide); VAC, temsirolimus, venaralbine, and cytoxan; doxorubicin and monoclonal antibody; AIM, monoclonal antibody, and gemcitabine. |

**Shown as number (percentage).**
